# Analysis of hematological parameters as prognostic markers for toxicity and survival of ^223^Radium treatment

**DOI:** 10.18632/oncotarget.24610

**Published:** 2018-03-05

**Authors:** Asha Leisser, Marzieh Nejabat, Markus Hartenbach, Reza Agha Mohammadi Sareshgi, Shahrokh Shariat, Gero Kramer, Michael Krainer, Marcus Hacker, Alexander R. Haug

**Affiliations:** ^1^ Department of Biomedical Imaging and Image-guided Therapy, Division of Nuclear Medicine, Medical University of Vienna, Vienna, Austria; ^2^ Department of Urology, Medical University of Vienna, Vienna, Austria; ^3^ Department of Oncology, Medical University of Vienna, Vienna, Austria; ^4^ FH Campus Wien, University of Applied Sciences, Department of Radiotechnology, Vienna, Austria

**Keywords:** ^223^Radium, hematotoxicity, radio-nuclide therapy, metastatic CRPC, adverse event

## Abstract

^223^Radium (^223^Ra) has emerged as treatment prolonging survival in patients with metastatic castration-resistant prostate cancer (CRPC). As ^223^Ra can cause hematotoxicity (HT), pre-existing hematopoiesis might influence the efficacy of ^223^Ra and the rate of hematotoxicity, but as to our knowledge such data has not been published yet, we retrospectively conducted an analysis on patients receiving ^223^Ra.

54 patients treated with ^223^Ra had a median survival of 67 weeks, which was significantly reduced in patients with pre-existing Hb toxicity (Tox) grade 2 (48 weeks *P* = 0.008) as compared to grade 1 (67 weeks) and normal levels of Hb (not reached); survival in patients with Plt Tox grade 1 was significantly reduced (44 weeks) as compared to normal Plt counts (71 weeks, *P* = 0.033). Patients with impaired hematopoiesis regarding Hb and Plts developed significantly more grade 3 and 4 HT (Hb < 10 g/dl: 42.9% [3/7] vs 10.6% [5/47], *P* < 0.001; Plt < 150 G/L: 28.6% [2/7] vs 6.4% [3/47], *P* = 0.002) and received significantly fewer treatment cycles (Hb <10 g/dl: 5.1 vs 5.8, *P* = 0.04; Plt < 150 G/L: 3.4 vs 5.6, *P* < 0.001). These results imply that pre-existing impaired hematopoiesis, in particular thrombocytopenia and anemia, before ^223^Ra therapy, is an important risk factor for worse outcome of treatment with ^223^Ra.

## INTRODUCTION

Prostate cancer is the second most common cancer in men worldwide with an estimated 1.1 million new cases diagnosed in 2012 [1]. Even after treatment with curative intent 27-53% patients experience disease recurrence [2, 3] with a significant portion of these patients deteriorating to develop castrate-resistant metastatic disease [4]. In this disease stage, chemotherapy (CHT) with docetaxel is one of the cornerstones of therapy [5, 6]. ^223^Radium (^223^Ra; Xofigo™, Alpharadin) has been approved for the treatment of bone metastases in patients with castration-resistant prostate cancer (CRPC) [7, 8], based on the data from the phase III, double-blind, randomized, clinical study of “alpharadin in symptomatic prostate cancer” [9] (ALSYMCA), which demonstrated an overall survival (OS) benefit of 30.5% (hazard ratio of 2.6), compared to best supportive care [9]. In the course of the ALSYMCA study mostly minor side effects, such as nausea, diarrhea, emesis and peripheral edema, but also grade 3/4 hematotoxicity (HT) were noted in up to 62% of patients [10], despite that mainly patients with sufficient hematopoietic reserve (Hb levels ≥ 10 g/dl) were included into the trial.

However, clinical use of a new therapeutic approach often differs in its patient selection than the controlled clinical trials that led to its approval. The therapeutic pressure is high and steadily increasing, leading to the treatment of patients with reduced or compromised hematopoietic reserves due to previously applied therapies, such as CHT and radiation therapy. To date, the impact of the grade of pre-therapeutic HT, in particular patients that present with a reduced hematopoietic reserve, on the ^223^Ra therapy efficacy has not been assessed, to our knowledge. We hypothesized that pre-therapeutic impaired hematopoiesis would affect ^223^Ra therapy oncologic efficacy and HT, leading to premature treatment termination and decreased overall survival (OS) in patients with metastatic CRPC (mCRPC).

## RESULTS

Patients’ demographics are listed in Table 1.

**Table 1:** Patient demographics

Variable	*n* (%)/value	Range (reference values and SI-unit)
**Mean/median age**	71/71	53–86 (years)
**ECOG**		
0	13 (24.1)	
1	22 (40.7)	
2	17 (31.5)	
3	2 (3.7)	
**Bone metastasis**		
<6	10 (18.5)	
6–20	16 (29.6)	
>20	21 (38.9)	
Super scan	7 (13.0)	
**Mean/median pre-therapeutic hematopoetic parameters**		
Hb start	11.7/12.1	8.3–14.2 (13.5–18.0 g/dL)
Leuk start	6.4/6.2	2.4–11.4 (4.0–10.0 G/L)
Plts start	243.8/227.5	81.0–534.0 (150–350 G/L)
**Mean/median post-therapeutic hematopoetic parameters**		
Hb end	11.0/11.7	6.9–13.8 (13.5–18.0 g/dL)
Leuk end	5.0/4.9	2.2–10.5 (4.0–10.0 G/L)
Plts end	200.8/198.0	44.0–458 (150–350 G/L)
**Previous treatments**		
RPE	43 (79.6)	
CHT	29 (53.7)	
Docetaxel + Cabazitaxel	5 (9.3)	
RT	39 (72.2)	
CHT + RT	23 (42.6)	
**Ongoing therapies**		
LHRH-Antagonist	20 (37.0)	
Enzalutamid/Bicalutamid	8 (14.8)	
Arbirateron	4 (7.4)	
GnRH-Antagonist	9 (16.7)	
None	13 (24.1)	

### Impact of pre-therapeutic AE on OS and number of treatment cycles

**Figure 1:**
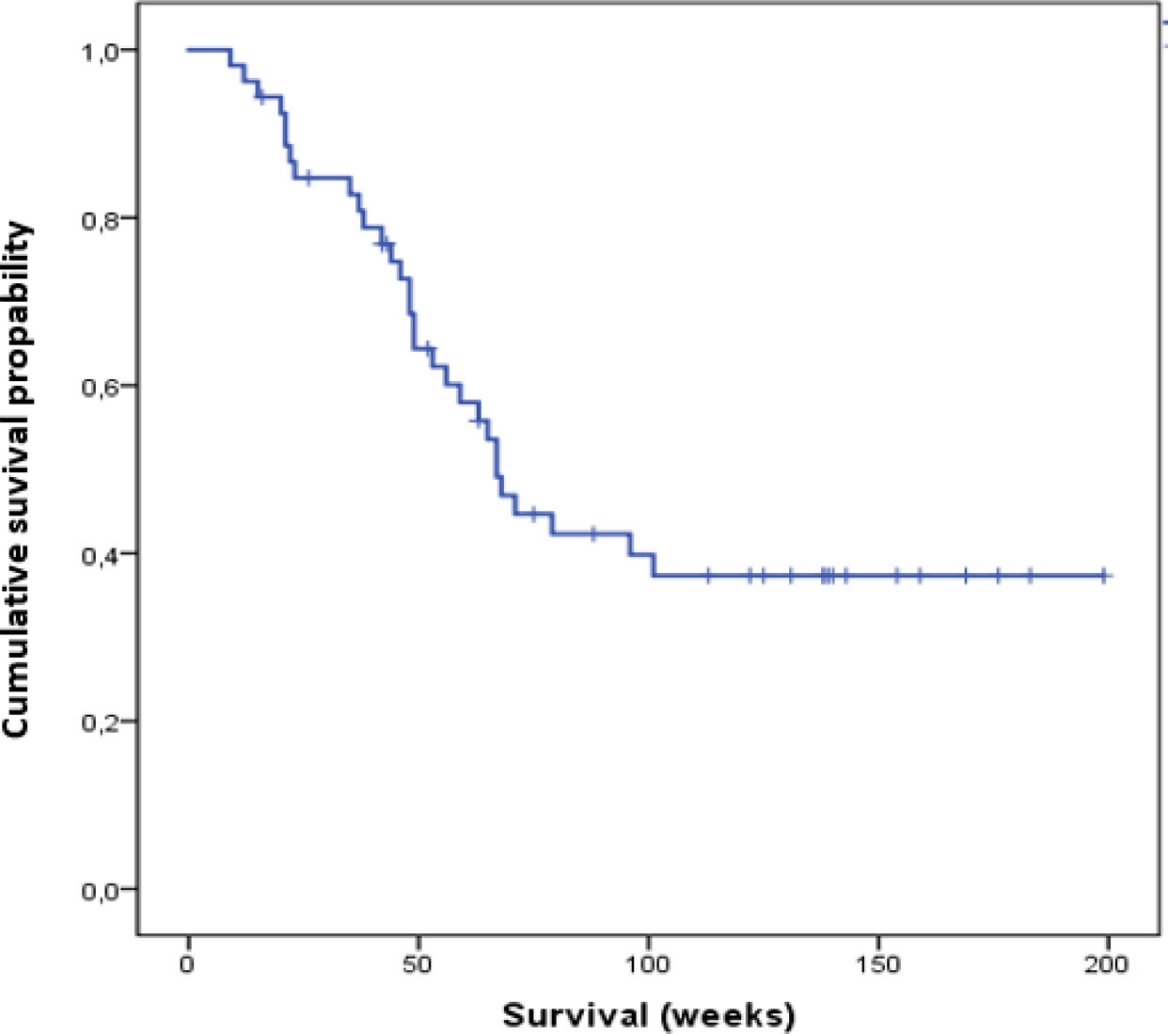
Cumulative overall survival of all patients analyzed in this study

Median OS in all patients was 67 weeks (range: 33.0–183.0 weeks) (Figure 1).

**Figure 2:**
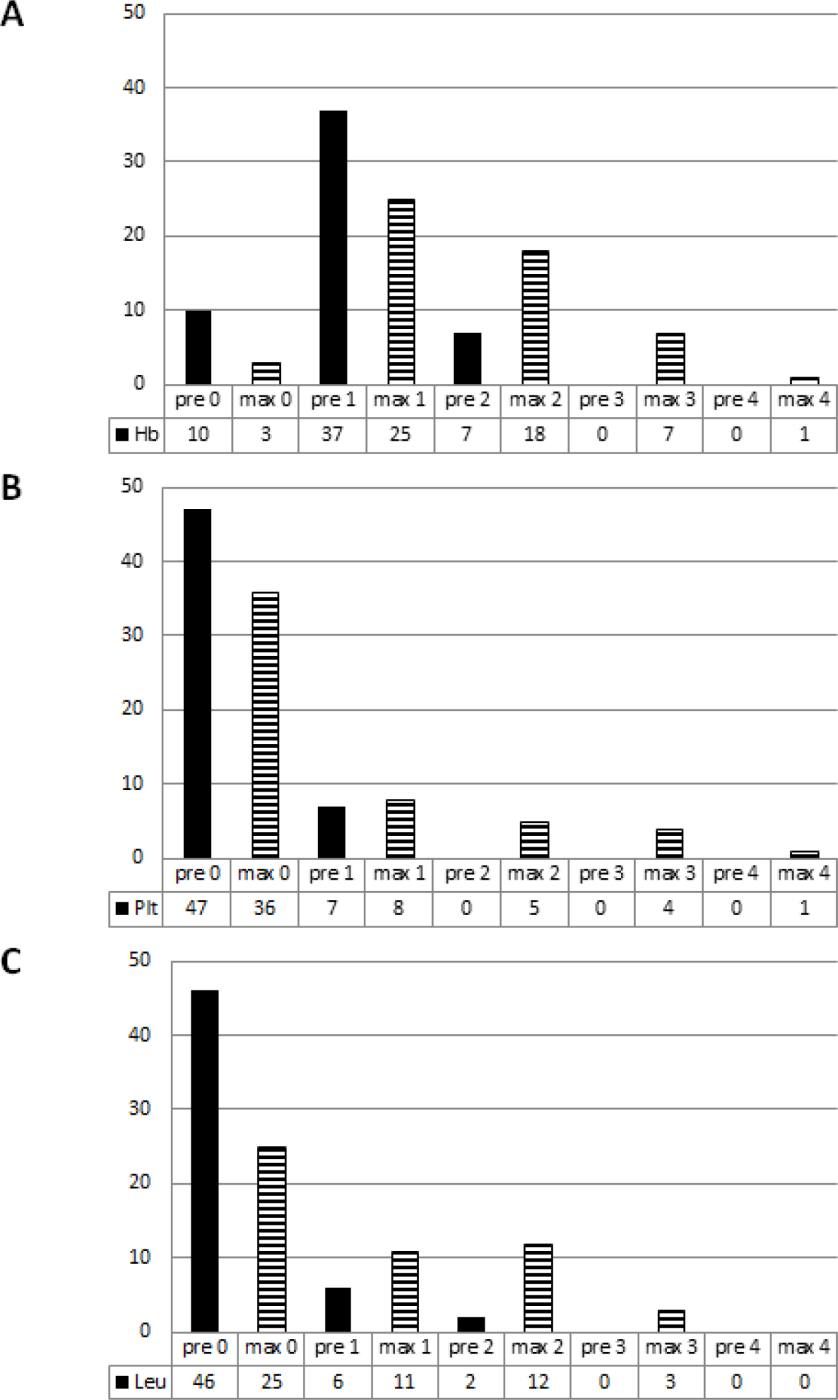
Number of pre-therapeutic (filled bars) and maximum AE grades (striped bars) with regard to the hemoglobin levels (**A**), the platelet (**B**) and the leukocyte count (**C**).

The majority of patients had pre-existing grade 1 and grade 2 HT regarding their Hb-levels (92.4%, Figure 2, Supplementary Table 1), while most patients presented with Plt (87%) and with Leuk counts (85%) within normal range.

Pre-therapeutic Leuk Tox had no significant impact on OS or number of treatment cycles (*P* = 0.09 and *P* = 0.12).

**Figure 3:**
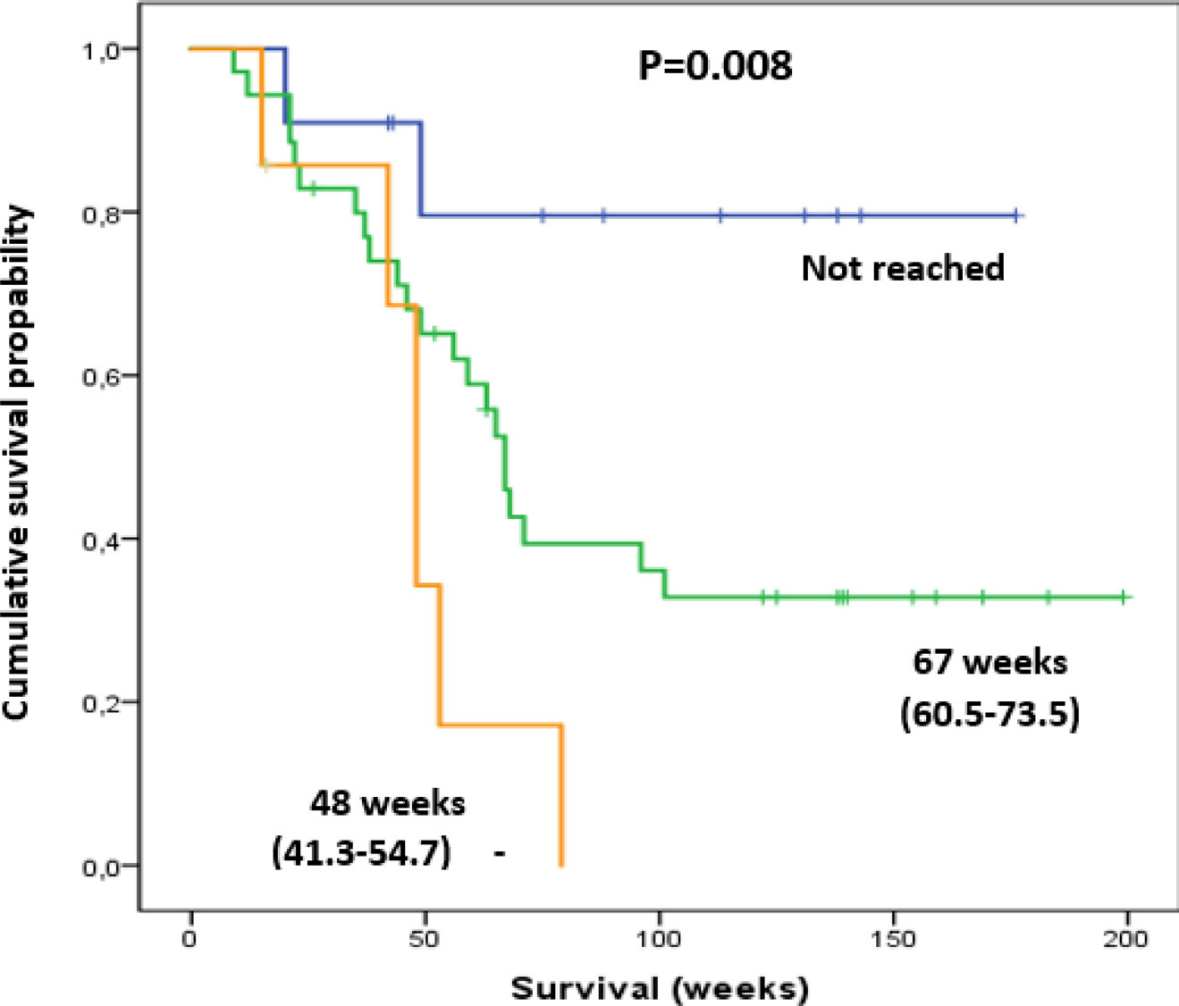
Patients with pre-therapeutic Hb Tox grade of 2 (orange line) had a shorter survival compared to those with Hb Tox grade of 0 (blue line) and 1 (green line) Shown in parentheses are ranges of survival in weeks (min-max).

Patients with pre-therapeutic Hb HT grade ≥2 (*n* = 7) had significantly shorter median survival (48.0 weeks) in comparison to those with HT grade 0 or 1 (grade 0: *n* = 10, median survival not reached; grade 1: *n* = 37, median survival 67 weeks; *P* = 0.008; Figure 3). These patients also received fewer treatment cycles (median number of cycles 5.1 vs 5.8, *P* = 0.04) and developed significantly more grade 3 or 4 HT (42.9% vs 13.5%, *P* < 0.001).

**Figure 4:**
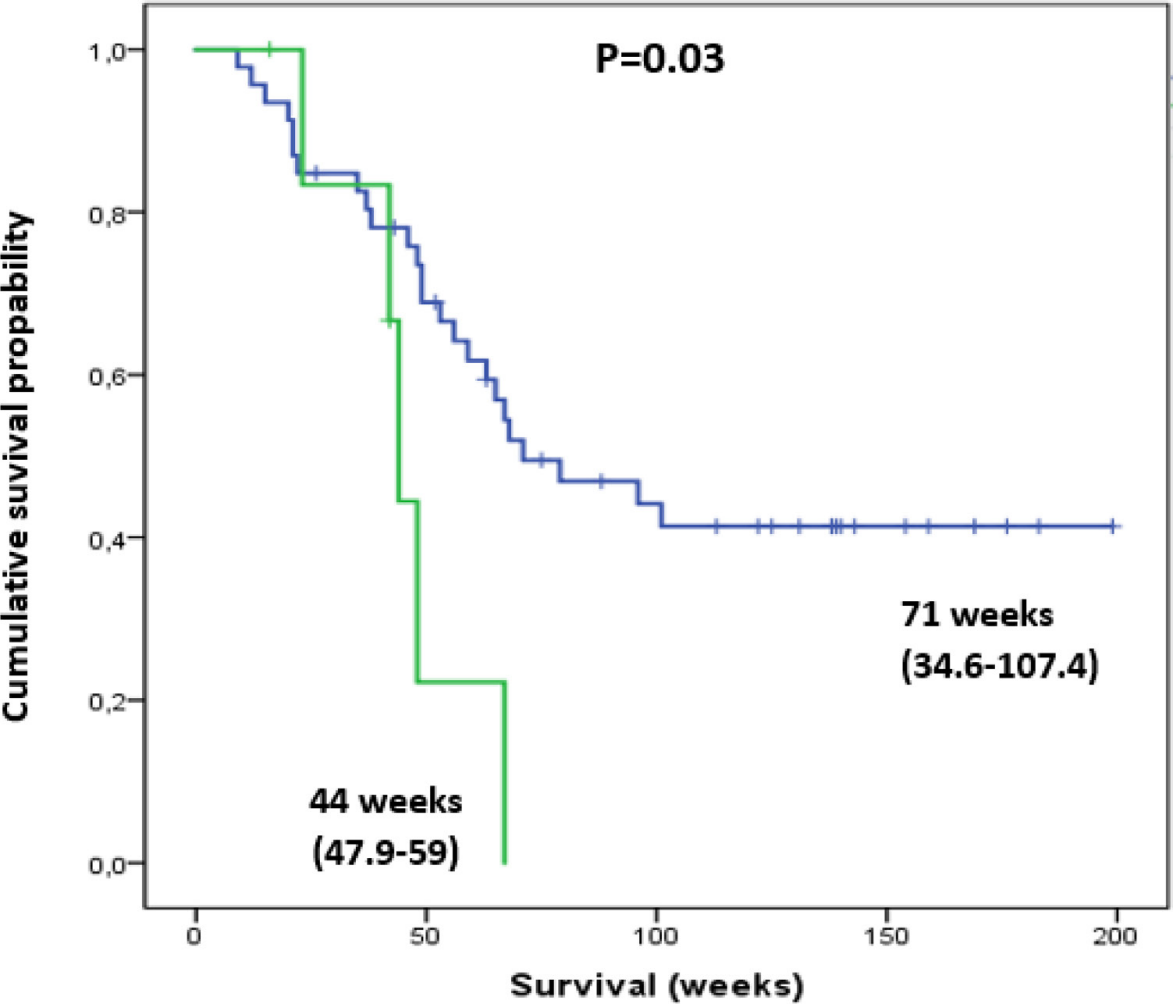
Thrombocytopenic patients with pre-therapeutic Plt Tox grade of 1 (green line) had a significantly shorter survival compared to those with normal Plt levels (blue line) Shown in parentheses are ranges of survival in weeks (min-max).

This effect was also present in patients demonstrating with thrombocytopenia. Grade 1 thrombocytopenia lead to reduced median OS (44 vs 71 weeks; *P* = 0.03) and the development of grade 3 or4 HT (28.6 vs 6.4%, *P* = 0.002) (Figure 4). Additionally, patients with grade 1 thrombocytopenia, received significantly fewer treatment cycles (median number of cycles 3.4 versus 5.6, *P* < 0.001).

### AEs according to pre-therapeutic Hb and Plt Tox grade

In total, 37/54 patients (68.5%) developed an AE or an increase in their AE grade (see details in Supplementary Table 1).

Patients with normal Hb levels (*n* = 10) developed no AEs grade 3, and developed grade1 (*n* = 6, 60%) and grade 2 (*n* = 1, 10%).

Patients with Hb level <10 g/dl (*n* = 7) developed AEs grade 3 and 4 in 42.9% of cases (*n* = 3/7), which was significantly more frequent (*P* < 0.001). For patients with normal Plt levels (*n* = 47) 2 patients (4%) developed AEs grade 3 (11 grade 1 [23.9%], 8 grade 2 [17.4%], 2 grade 3 [4.3%]), while patients with decreased levels of Plt (*n* = 8) developed grade 3 AEs in 12.5% (*n* = 1; 6 grade 2 [75.0%]; *P* = 0.002).

In our cohort of 54 patients, 7 (13.0 %) were diagnosed with diffuse metastatic bony disease (super scan) before treatment initiation with ^223^Radium as defined in the materials and methods. Only 29 (53.7%) underwent chemotherapy with taxanes (median number of cycles: 6) and 39 (72.2%) radiation therapy; 23 (42.6%) patients received both.

However, neither extent of bone metastases, nor previous chemo- or radiation therapy were significantly associated with the number of ^223^Ra cycles and hematotoxicity (Table 2).

**Table 2:** Significance of risk factors on OS, number of ^223^Ra treatment cycles and HT (respective *P* values are given)

	<20 vs >20 bone mets	CHT	RT
**OS**	0.32	0.77	0.65
**number of ^223^Ra cycles**	0.14	0.44	0.12
**HT**	0.32	0.8	0.32

## DISCUSSION

In anticipation of its shorter therapeutic range coupled with a very high linear energy transfer, the alpha particle emitter ^223^Ra potentially causes fewer hematologic AE in comparison to the so far utilized beta-emitting radionuclides [11]. Up to now, ^223^Ra safety and efficacy has mainly been studied in the patient cohort of ALSYMPCA and the named access program. These patients were all reported to have sufficient hematopoietic function, both in patients that received docetaxel before radionuclide therapy and the ones that were docetaxel naïve [9]. Yet the clinical truth is - as shown with this retrospective analysis- that the majority of patients present with impaired hematopoiesis. The main aim of this retrospective analysis was to investigate further into the treatment efficacy and safety of ^223^Ra therapy in patients with a reduced hematopoietic reserve.

As expected, patients with a good hematopoietic reserve regarding their hemoglobin levels seldom developed HT and demonstrated prolonged survival in comparison to the remaining patients. These results comply with the findings of Hoskin *et al.* in ALSYMPCA [8].

However, in the cohort studied in this retrospective analysis it was observed that if hematopoiesis was reduced, especially in regard to hemoglobin levels and platelet counts, a significantly higher number of AEs and shorter overall survival occurred. This partially can be explained by the fact that pre-therapeutically induced anemia (Hb < 10 g/dl) and thrombocytopenia (platelet counts < 150 G/L) progressed during ^223^Ra treatment. These circumstances gave cause to interruption of ^223^Radium treatment significantly more often, which consequently might reduce therapy efficacy and may even give rise to therapy resistance [12, 13]. Another explanation might be a more advanced tumor stage in these patients with regard to bone marrow involvement, which then impairs hematopoiesis. These results imply that for these patients treatment with ^223^Ra may be less beneficial in terms of OS and quality of life. Our findings also go in accordance with a recent study published by McKay *et al.*, who showed that hemoglobin levels and white blood counts higher than at the lower limit of normal range were factors associated with therapy completion [14].

Previously conducted chemo-and radiation therapy are known risk factors for reducing hematopoiesis and developing HT which is associated with a poorer quality of life, prognosis and overall survival [15, 16]. In particular, anemia seems to play a crucial role [17, 18]. Nevertheless, these standards of care treatment options are indispensable for patients with CRPC. As demonstrated with this study, the border between beneficial therapy and causing relevant side effects is very narrow. Therefore, an accordingly strict patient selection for ^223^Ra treatment is essential to guarantee the best possible and most effective therapeutic patient care in this patient population. This is underlined by the finding of a very favorable survival and toxicity profile in patients with regular or only mildly impaired (grade 1) hematopoiesis treated with ^223^Ra in the presented study.

For future perspective and based on the good safety and efficacy of ^223^Ra as published by Hoskin *et al.* [8] might be intriguing to use this therapeutic option earlier or as an add-on to current treatment regimen. Here, further studies are currently ongoing.

To conclude, impaired hematopoiesis before ^223^Radium therapy was significantly related to shorter survival and the development of severe HT during therapy. This mainly affected hemoglobin values and platelet counts, thus leading to increasing numbers of premature termination of ^223^Ra treatments and consequentially to a reduced therapy efficacy. Contrary, patients with normal hematopoiesis had a favorable outcome.

## MATERIALS AND METHODS

### Study design and patient population

This retrospective, single-center study was approved by an institutional review board and the need for written informed consent was waived. Patient history of 54 consecutive males with verified mCRPC and symptomatic bone metastases (mean age: 71 years ± 9; range: 54–88 years), who were referred for treatment with ^223^Ra between December 2013 and December 2015 were reviewed.

The recommendation for treatment with ^223^Ra was given by an interdisciplinary tumor board, consisting of urologists, radiologists, oncologist, pathologists, radiation oncologists as well as a nuclear medicine physician. Each treatment cycle consisted of an intravenous injection of 55 kBq ^223^Ra per kg of body weight of the radiopharmaceutical diluted in 5 ml isotonic sodium chloride solution.

In our cohort 4 patients (14.8%) received 1 to 4, and 46 patients (85.2%) received 5 to 6 cycles of ^223^Ra.

### Inclusion criteria included

(a) histologically verified PCa, (b) conducted treatments such as RPE and/or RT, (c) verification of metastatic disease with bone involvement by bone scintigraphy and CT and/or MRI, (d) completed ^223^Ra therapy at time of evaluation, (e) complete history of laboratory results of regular check-ups and interim treatment control time points, (f) exclusion of visceral metastases and lymph node metastases larger than 3 cm.

### Exclusion criteria

For ^223^Ra therapy included alterations of the complete blood count (Hb > 8 mg/dl, Plt > 50 G/L, Leuk > 1G/L), which require therapeutical countermeasures such as blood transfusions, and that were caused by a deficiency of co-factors and substrates (e.g. iron, vitamin B12 and folic acid), secondary due to other chronic diseases (e.g. renal insufficiency with indication for dialysis, parasite infections, hemolytic anemia, autoimmune associated syndromes such as systemic lupus erythematodes or idiopathic thrombocytopenic purpura) or resulting from hereditary effects (e.g. thalassemia, elliptocytosis).

Routine treatment monitoring consisted of a complete laboratory work-up before each treatment cycle as well as a CT scan and bone scintigraphy before the first and after the third cycle as well as after completion of ^223^Ra therapy, respectively. Patients were seen at least once a month for follow-up at which point they received a full laboratory work-up during ^223^Ra therapy and at least 6 months after. For determination of OS patients were followed-up until November 2017. Median follow-up after ^223^Ra therapy was 126.4 weeks (range: 33.0–183.0 weeks).

### Evaluation of adverse events and collection of study parameters

Grading of the hematologic AEs was conducted complying with the CTCAE recommendations version 5.0 [19]. In order to classify pre-therapeutic HT, hemoglobin-levels, leukocyte and platelet counts before the first treatment cycle with ^223^Ra were graded from 1–5 (see Table 1). Following the same approach, grading AEs in the course of and after the entire ^223^Ra therapy, taking into account the blood results of interim check-ups and follow-ups, was conducted. Those patients that demonstrated normal blood counts were assigned grade 0. Additionally, we distinguished with regard to the extent of bone metastases based on bone scintigraphy by dividing into subgroups (<6, 6–20, >20 and super scan).

### Statistics

Statistical analysis was executed with the program IBM SPSS 22 and involved descriptive statistics, correlations with Spearman-Rho analyses, ANOVA analysis as well as Kruskal–Wallis test, testing for significant differences. Additionally, the influence of OS was determined by executing Kaplan–Meier analyses with log-rank test. All tests are two-sided and the significance level was set at *p* < 0.05.

## SUPPLEMENTARY MATERIALS TABLES



## Abbreviations

metastatic CRPC: metastatic castration-resistant prostate cancer
AE: adverse event
ECOG: Eastern Co-operative of Oncology Group
Hb: hemoglobin
Leuk: leukocytes
Plts: platelets
PSA: prostate serum antigen
HT: hematotoxicity
^223^Ra: Radium 223
CHT: chemotherapy
RPE: radical prostatectomy
RT: radiation therapy
