# Supplementary material for: Analysis of hematological parameters as prognostic markers for toxicity and survival of 223Radium treatment
Source: Oncotarget. 2018 Mar 5;9(22):16197–204. doi: 10.18632/oncotarget.24610 (PMC5882327; doi:10.18632/oncotarget.24610)
Supplement: Supplementary file 1 [file oncotarget-09-16197-s001.pdf]

Analysis of hematological parameters as prognostic markers for toxicity and survival of ^{223}Radium treatment

SUPPLEMENTARY MATERIALS

Supplementary Table 1: AE grades in regards to pre-therapeutic hemoglobin-levels, platelet- and leukocyte counts

	Hemoglobin		Platelets		Leukocytes	
	Total number	%	Total number	%	Total number	%
**Starting Grade 0**	*n* = 10		*n* = 47		*n* = 46	
Grade 0	3	30.0	36	76.6	25	54.3
Grade 1	6	60.0	6	12.8	11	23.9
Grade 2	1	10.0	2	4.3	8	17.4
Grade 3	0	0.0	2	4.3	2	4.3
Grade 4	0	0.0	1	2.1	0	0.0
All events > initial AE grade	*7*	*70.0*	*11*	*23.4*	*21*	*45.7*
**Starting Grade 1**	*n* = 37		*n* = 7		*n* = 6	
Grade 0	0	0.0	0	0.0	0	0.0
Grade 1	18	48.6	2	28.6	1	16.7
Grade 2	14	37.8	3	42.8	5	83.3
Grade 3	4	10.8	2	28.6	0	0.0
Grade 4	1	2.7	0	0.0	0	0.0
All events > initial AE grade	*19*	*51.4*	*5*	*71.4*	*5*	*83.3*
**Starting Grade 2**	*n* = 7		*n* = 0		*n* = 2	
Grade 0	0	0.0	0	0.0	0	0.0
Grade 1	1	14.3	0	0.0	0	
Grade 2	3	42.9	0	0.0	1	50.0
Grade 3	3	42.9	0	0.0	1	50.0
Grade 4	0	0.0	0	0.0	0	0.0
All events > initial AE grade	*3*	*42.9*	*0*	*0.0*	*1*	*50.0*
